# Age and onset timing of Raynaud’s phenomenon and first non-Raynaud symptom as prognostic factors in systemic sclerosis: a retrospective analysis from the Italian national multicenter Systemic Sclerosis Progression INvestiGation registry of the Italian Society for Rheumatology (SPRING-SIR)

**DOI:** 10.1177/1759720X251410243

**Published:** 2026-02-05

**Authors:** Silvia Peretti, Cosimo Bruni, Francesco Bonomi, Rossella De Angelis, Gianluigi Bajocchi, Dilia Giuggioli, Martina Orlandi, Giovanni Zanframundo, Roberta Foti, Elisa Visalli, Giovanna Cuomo, Alarico Ariani, Edoardo Rosato, Gemma Lepri, Francesco Girelli, Valeria Riccieri, Elisabetta Zanatta, Silvia Laura Bosello, Ilaria Cavazzana, Francesca Ingegnoli, Maria De Santis, Fabio Cacciapaglia, Giuseppe Murdaca, Giuseppina Abignano, Giorgio Pettiti, Alessandra Della Rossa, Maurizio Caminiti, Annamaria Iuliano, Giovanni Ciano, Lorenzo Beretta, Gianluca Bagnato, Ennio Lubrano, Ilenia De Andres, Luca Idolazzi, Marta Saracco, Cecilia Agnes, Corrado Campochiaro, Giacomo De Luca, Edoardo Cipolletta, Marco Fornaro, Federica Lumetti, Amelia Spinella, Luca Magnani, Veronica Codullo, Carlo Iandoli, Antonietta Gigante, Greta Pellegrino, Erika Pigatto, Maria Grazia Lazzaroni, Enrico De Lorenzis, Gianna Angela Mennillo, Marco Di Battista, Giuseppa Pagano Mariano, Federica Furini, Licia Vultaggio, Simone Parisi, Clara Lisa Peroni, Gerolamo Bianchi, Enrico Fusaro, Gian Domenico Sebastiani, Marcello Govoni, Salvatore D’Angelo, Franco Cozzi, Franco Franceschini, Serena Guiducci, Lorenzo Dagna, Andrea Doria, Carlo Salvarani, Maria Antonietta D’Agostino, Florenzo Iannone, Marco Matucci-Cerinic, Clodoveo Ferri, Silvia Bellando Randone

**Affiliations:** Department of Experimental and Clinical Medicine, University of Florence, Florence, Italy; Division of Rheumatology, Department of Medical and Geriatric specialties, Careggi University Hospital, University of Florence, Florence, Italy; Department of Rheumatology, University Hospital Zurich, University of Zurich, Zurich 8091, Switzerland; Department of Experimental and Clinical Medicine, University of Florence, Florence, Italy; Department of Experimental and Clinical Medicine, University of Florence, Florence, Italy; Division of Rheumatology, Department of Medical and Geriatric specialties, Careggi University Hospital, University of Florence, Florence, Italy; Rheumatology Unit, Department of Clinical and Molecular Sciences, Polytechnic University of Marche, Ancona, Italy; Rheumatology Unit, S. Maria Hospital-USL, IRCCS Institute, Reggio Emilia, Italy; Department of Medical and Surgical Sciences for Children and Adults, University Hospital of Modena and Reggio Emilia School of Medicine, Modena, Italy; Department of Medical and Surgical Sciences for Children and Adults, University Hospital of Modena and Reggio Emilia School of Medicine, Modena, Italy; Department of Internal Medicine and Therapeutics, Università di Pavia, Pavia, Italy; Division of Rheumatology, Fondazione IRCCS Policlinico San Matteo, Pavia, Italy; Rheumatology Unit, AOU Policlinico San Marco, Catania, Italy; Rheumatology Unit, AOU Policlinico San Marco, Catania, Italy; Department of Precision Medicine, University of Campania—Luigi Vanvitelli University, Naples, Italy; Department of Medicine, Internal Medicine and Rheumatology, Azienda Ospedaliero—Universitaria di Parma, Parma, Italy; Department of Translational and Precision Medicine, Sapienza University of Rome, Rome, Italy; Department of Experimental and Clinical Medicine, University of Florence, Florence, Italy; Division of Rheumatology, Department of Medical and Geriatric specialties, Careggi University Hospital, University of Florence, Florence, Italy; Department of Medicine, Rheumatology Unit, Ospedale GB Morgagni—L Pierantoni, Forlì, Italy; Department of Internal Medicine, Anesthesiology and Cardiovascular Sciences, Sapienza University of Rome, Rome, Italy; Department of Rheumatology, University of Padua, Padova, Italy; Rheumatology Division, Catholic University of the Sacred Heart, Fondazione Policlinico Universitario A. Gemelli—IRCCS, Rome, Italy; Rheumatology and Clinical Immunology, ASST Spedali Civili of Brescia, Brescia, Italy; Department of Clinical and Experimental Sciences, University of Brescia, Brescia, Italy; Clinica Reumatologica, Dipartimento di Reumatologia e Scienze Mediche, ASST Gaetano Pini-CTO, Dipartimento di Scienze Cliniche e di Comunità, Dipartimento di Eccellenza, Università degli Studi di Milano, Milan, Italy; Department of Biomedical Sciences, Humanitas University, Pieve Emanuele—Milan and Research Hospital, Milan, Italy; Rheumatology Unit, Department of Precision and Regenerative Medicine—Ionian Area, University of Bari “Aldo Moro,” Bari, Italy; Rheumatology Service, Internal Medicine Unit “F. Miulli” General Hospital, Acquaviva delle Fonti, Bari, Italy; Department of Medicine and Surgery, LUM “G. De Gennaro,” Casamassima, Bari, Italy; Department of Internal Medicine, University of Genoa and Allergology, and Clinical Immunology Unit, Ospedale San Bartolomeo, Sarzana, Italy; Rheumatology Unit, Department of Health Science, San Carlo Hospital, University of Basilicata, Potenza, Italy; Rheumatology Unit ASO S., Croce e Carle Hospital, Cuneo, Italy; Department of Rheumatology, University of Pisa, Pisa, Italy; Departmental Rheumatology Unit, Grande Ospedale Metropolitano, Reggio Calabria, Italy; Rheumatology Unit, San Camillo–Forlanini Hospital, Rome, Italy; Local Health Department, Hospital of Ariano Irpino, Ariano Irpino, Italy; Referral Center for Systemic Autoimmune Diseases, Fondazione IRCCS Ca’ Granda, Ospedale Maggiore Policlinico di Milano, Milan, Italy; Department of Clinical and Experimental Medicine, University of Messina, Messina, Italy; Department of Rheumatology, University of Molise, Campobasso, Italy; Rheumatology Unit, Azienda Ospedaliera di Rilievo Nazionale ed Alta Specializzazione Garibaldi, Catania, Italy; Rheumatology Unit, Department of Medicine, University of Verona, Verona, Italy; Rheumatology Unit, Mauriziano-Umberto I Hospital, Turin, Italy; Division of Rehabilitation, Department of Medicine, Torino, ASL TO5, Carmagnola, Italy; IRCCS San Raffaele Scientific Institute, Unit of Immunology, Rheumatology, Allergy and Rare Diseases, & Inflammation, Fibrosis and Ageing initiative, Milan, Italy; Vita-Salute San Raffaele University, Milan, Italy; IRCCS San Raffaele Scientific Institute, Unit of Immunology, Rheumatology, Allergy and Rare Diseases, & Inflammation, Fibrosis and Ageing initiative, Milan, Italy; Vita-Salute San Raffaele University, Milan, Italy; Rheumatology Unit, Department of Clinical and Molecular Sciences, Polytechnic University of Marche, Ancona, Italy; Rheumatology Unit, Department of Precision and Regenerative Medicine—Ionian Area, University of Bari “Aldo Moro,” Bari, Italy; Department of Medical and Surgical Sciences for Children and Adults, University Hospital of Modena and Reggio Emilia School of Medicine, Modena, Italy; Department of Medical and Surgical Sciences for Children and Adults, University Hospital of Modena and Reggio Emilia School of Medicine, Modena, Italy; Rheumatology Unit, S. Maria Hospital-USL, IRCCS Institute, Reggio Emilia, Italy; Department of Internal Medicine and Therapeutics, Università di Pavia, Pavia, Italy; Division of Rheumatology, Fondazione IRCCS Policlinico San Matteo, Pavia, Italy; Department of Precision Medicine, University of Campania—Luigi Vanvitelli University, Naples, Italy; Department of Translational and Precision Medicine, Sapienza University of Rome, Rome, Italy; Department of Rheumatology, IRCCS Ospedale Galeazzi—Sant’Ambrogio, Milan, Italy; Dipartimento di Scienze Biomediche e Cliniche, Università degli Studi di Milano, Milan, Italy; Department of Medicine, Villa Salus Hospital, Mestre, Italy; Rheumatology and Clinical Immunology, ASST Spedali Civili of Brescia, Brescia, Italy; Department of Clinical and Experimental Sciences, University of Brescia, Brescia, Italy; Rheumatology Division, Catholic University of the Sacred Heart, Fondazione Policlinico Universitario A. Gemelli—IRCCS, Rome, Italy; Rheumatology Unit, Department of Health Science, San Carlo Hospital, University of Basilicata, Potenza, Italy; Department of Rheumatology, University of Pisa, Pisa, Italy; Departmental Rheumatology Unit, Grande Ospedale Metropolitano, Reggio Calabria, Italy; Department of Medicine, Villa Salus Hospital, Mestre, Italy; Rheumatology Unit, Department of Medical Sciences, University of Ferrara and Azienda Ospedaliera—Universitaria S. Anna, Ferrara, Italy; Rheumatology Unit, Azienda Ospedaliera Universitaria Città Della Salute e della Scienza di Torino, Turin, Italy; Rheumatology Unit, Azienda Ospedaliera Universitaria Città Della Salute e della Scienza di Torino, Turin, Italy; Rheumatology Unit, Department of Medical Specialities, Local Health Trust 3, Genoa, Italy; Rheumatology Unit, Azienda Ospedaliera Universitaria Città Della Salute e della Scienza di Torino, Turin, Italy; Rheumatology Unit, San Camillo–Forlanini Hospital, Rome, Italy; Rheumatology Unit, Department of Medical Sciences, University of Ferrara and Azienda Ospedaliera—Universitaria S. Anna, Ferrara, Italy; Rheumatology Unit, Department of Health Science, San Carlo Hospital, University of Basilicata, Potenza, Italy; Department of Medicine, Villa Salus Hospital, Mestre, Italy; Rheumatology and Clinical Immunology, ASST Spedali Civili of Brescia, Brescia, Italy; Department of Clinical and Experimental Sciences, University of Brescia, Brescia, Italy; Department of Experimental and Clinical Medicine, University of Florence, Florence, Italy; Division of Rheumatology, Department of Medical and Geriatric specialties, Careggi University Hospital, University of Florence, Florence, Italy; IRCCS San Raffaele Scientific Institute, Unit of Immunology, Rheumatology, Allergy and Rare Diseases, & Inflammation, Fibrosis and Ageing initiative, Milan, Italy; Vita-Salute San Raffaele University, Milan, Italy; Department of Rheumatology, University of Padua, Padova, Italy; Department of Medical and Surgical Sciences for Children and Adults, University Hospital of Modena and Reggio Emilia School of Medicine, Modena, Italy; Rheumatology Division, Catholic University of the Sacred Heart, Fondazione Policlinico Universitario A. Gemelli—IRCCS, Rome, Italy; Rheumatology Unit, Department of Precision and Regenerative Medicine—Ionian Area, University of Bari “Aldo Moro,” Bari, Italy; IRCCS San Raffaele Scientific Institute, Unit of Immunology, Rheumatology, Allergy and Rare Diseases, & Inflammation, Fibrosis and Ageing initiative, Milan, Italy; Vita-Salute San Raffaele University, Milan, Italy; Department of Medical and Surgical Sciences for Children and Adults, University Hospital of Modena and Reggio Emilia School of Medicine, Modena, Italy; Rheumatology Clinic “Madonna dello Scoglio” Crotonei, Crotone, Italy; Department of Experimental and Clinical Medicine, University of Florence, Florence, Italy; Division of Rheumatology, Department of Medical and Geriatric specialties, Careggi University Hospital, University of Florence, Florence, Italy

**Keywords:** disease onset, non-Raynaud’s symptoms, Raynaud’s phenomenon, risk assessment, systemic sclerosis

## Abstract

**Background:**

The sequence and temporal relationship between Raynaud’s phenomenon (RP) and the first non-Raynaud’s sign/symptom (NRP) in systemic sclerosis (SSc) have been partially investigated.

**Objectives:**

To evaluate whether the mode and ages of clinical onset are associated with disease endotype and survival in SSc.

**Design:**

We included SSc patients from the Systemic sclerosis Progression INvestiGation registry of the Italian Society of Rheumatology (SPRING-SIR) registry in a cohort study, with post hoc cross-sectional and longitudinal analysis.

**Methods:**

Patients were grouped based on age-RP and age-NRP quartiles. Additionally, categories were defined based on mode of onset: RP group—RP onset at least 1 year before NRP; Simultaneous group—RP onset within the same year of NRP; NRP group—RP onset after at least 1 year after NRP. Comparisons were made using Chi-square and ANOVA tests. Logistic, linear, and multinomial regression models were applied to assess associations, while Kaplan–Meier curves and Cox regression were used to assess mortality.

**Results:**

A total of 1748 patients were eligible: 682 (39.0%) in the RP group, 1026 (58.8%) in the simultaneous group, and 39 (2.2%) in the NRP group. A higher prevalence of anti-centromere antibodies was found In the RP group, while the simultaneous group had more diffuse cutaneous SSc (dcSSc), anti-topoisomerase-I antibodies, and higher Rodnan’s skin score (mRSS). The NRP group presented higher prevalence of pulmonary arterial hypertension. On logistic regression, the simultaneous group was associated with a higher prevalence of dcSSc compared to the RP group (odds ratio, 1.491, 95% confidence interval (CI): 1.032–2.154). Younger age at RP onset was associated with lower systolic pulmonary artery pressure and mRSS. In 943 patients with available follow-up (median 24 months), the simultaneous group had higher mortality compared to the RP group (hazard ratio, 1.975, 95% CI: 1.002–3.893).

**Conclusion:**

The timing of RP and NRP onset may help define SSc endotype and survival. Patients with simultaneous RP-NRP onset have more severe disease features and higher mortality risk, emphasizing the relevance of onset timing in disease stratification.

## Plain language summary

How systemic sclerosis starts impacts how the disease will behave

Systemic sclerosis (SSc) is a rare autoimmune disease that affects the skin and internal organs. In most cases the disease starts with Raynaud’s phenomenon (RP), a discolouring of the fingers in reaction to cold temperatures; some other patients may experience different symptoms first, such as joint pain, skin changes or shortness of breath. Understanding which symptom appears first, and at what age, may help doctors identify patients who are more likely to develop severe disease. This study included 1,748 SSc patients enrolled in the Italian SPRING-SIR registry, divided patients into three groups: those who first developed RP (RP group), those who developed RP and other symptoms in the same year (Simultaneous), and those who developed non-RP symptoms first (NRP group). We found that patients in the RP group were younger at disease onset and often had milder disease. Patients in the Simultaneous group were more likely to develop diffuse skin disease, lung complications, and had a higher risk of death. Patients in the NRPgroup were rare but often had aggressive involvement of heart and lung. Overall, our results show that the timing of RP and non-RP symptoms can help predict disease course and survival in SSc. These findings may help doctors recognize higher-risk patients earlier and provide closer monitoring and tailored treatments.

## Introduction

Systemic sclerosis (SSc) is a rare autoimmune disease characterized by widespread vascular damage, immune dysregulation, and fibrotic changes affecting the skin and internal organs.^1,2^ The disease primarily affects females, with a female-to-male ratio ranging from 3:1 to 8:1, and shows two incidence peaks in the age groups 45–54 and 65–74 years based on race and sex.^3^

Among its diverse clinical presentations, Raynaud’s phenomenon (RP) occurs in over 95% of SSc patients. It is the commonest initial symptom in SSc patients and generally precedes the onset of the disease by several years,^4–7^ especially in the limited cutaneous SSc subset (lcSSc). Conversely, patients exhibit other non-Raynaud’s signs or symptoms (NRP) soon after the development of RP in the diffuse cutaneous subset (dcSSc).^8,9^ A short interval (⩽1 year) between RP and the appearance of NRP symptoms has been previously associated with more aggressive disease endotype, including a higher prevalence of anti-topoisomerase I antibodies (ATA), dcSSc, and a lower 10-year survival rate. This association was demonstrated in two large Italian cohort studies conducted by Ferri et al.,^10,11^ which highlighted the prognostic significance of symptom onset timing in SSc. More recently, De Angelis et al.^12^ analyzed the SPRING registry focusing on the sine scleroderma subset (ssSSc), and demonstrated that the timing from RP onset to diagnosis significantly differed across SSc skin subsets, being longer in ssSSc compared to both lcSSc and dcSSc.^12^ These findings underline the importance of onset timing as a prognostic factor, but evidence from large cohorts stratifying patients by RP- or NRP-onset is lacking.

Furthermore, other studies have identified factors associated with worse outcomes in SSc, such as male sex, dcSSc, interstitial lung disease (ILD), and older age at disease onset.^10,11,13–19^ The relationship between age at RP onset, the age of onset of the first non-Raynaud’s phenomenon sign or symptom (NRP), and their timing (i.e., whether RP occurs before, after, or simultaneously with NRP) remains partially underexplored, although initial evidence suggests that NRP symptoms/signs at onset are independent predictors of more aggressive disease features such as visceral involvement, and worse survival compared to RP at onset.^20^

The primary aim of our investigation was to assess whether the mode of SSc onset (RP vs NRP) along with the age at their onset are associated with the disease endotype and the prognosis in SSc patients. This data would further underline the importance of stratifying SSc patients based on RP and NRP patterns of onset, aiming to improve risk assessment and support the development of personalized management strategies.^20^

## Methods

### Study design

We performed a cohort study, including a post hoc, cross-sectional analysis to evaluate the association between the mode of disease onset and clinical endotype in patients with SSc. In addition, we conducted an exploratory longitudinal analysis to compare survival rates across patients presenting with different onset patterns. The reporting of this study conforms to the “Strengthening the Reporting of Observational Studies in Epidemiology (STROBE)” statement.^21^

### Data source

We extracted relevant data from the Systemic sclerosis Progression INvestiGation registry of the Italian Society of Rheumatology (SPRING-SIR). SPRING-SIR is a multicenter, national cohort study promoted by the Italian Society of Rheumatology (SIR) which involved 38 referral centers across Italy, with expertise in the diagnosis and management of SSc. The study was conducted in accordance with the Declaration of Helsinki. The protocol was approved by the coordinating ethics committee of AOU Careggi, Florence (reference number OSS 15.010 AOU Careggi, Firenze, Italy), and by the ethics committees of all participating centers. All participants had previously provided written informed consent at the time of enrollment in the SPRING-SIR registry; therefore, no additional consent was required for this retrospective analysis.

For each patient, demographic, clinical, and immunological data were collected in line with the nature of the registry, with follow-up up to 5 years.^22,23^

### Inclusion and exclusion criteria

Patients were eligible for inclusion in our analysis if they:

- were enrolled in the SPRING-SIR registry;
- were aged ⩾18 years at the time of registry enrollment;
- fulfilled the 2013 American College of Rheumatology/European League Against Rheumatism (EULAR) classification criteria for systemic sclerosis;
- had available data on the year of onset for both RP and the first NRP sign or symptom;

otherwise excluded from our study.

### Exposure

Patients were categorized into four groups both for age-RP and age-NRP, based on the quartiles of the distribution in the total population. Since age at RP onset and age at NRP onset follow different distributions within the cohort, the resulting quartile groups differed in terms of age range. This approach ensured that each group contained a similar number of patients, allowing for balanced comparisons across categories. Additionally, patients were divided into three groups based on RP onset patterns: (1) RP group: RP onset at least 1 year before NRP sign/symptom; (2) Simultaneous group: RP onset within the same calendar year of NRP sign/symptom (therefore corresponding to a maximum interval of 12); and (3) NRP group: RP onset after at least 1 year after NRP sign/symptom.

### Outcome

The primary outcome of this study was to assess the association between the mode of onset defined as the temporal sequence between RP and NRP and the clinical endotype of SSc. As an exploratory outcome, the association between mode of onset and mortality was additionally tested.

### Covariates

For all patients, demographic and clinical data were collected at the baseline visit. While the age of onset of RP was patient-reported, the first NRP sign or symptom was documented by the physician, based on both patient report and reviewed clinical documentation. ILD was defined as the presence of interstitial pattern changes on high resolution computed tomography (HRCT) such as reticulation, traction bronchiectasis, pleural irregularities, lung volume loss, and honeycombing. Pulmonary arterial hypertension (PAH) was defined as a mean pulmonary artery pressure (PAPm) >20 mmHg on right heart catheterization.^24^ Cardiopulmonary manifestations encompassed ILD, PAH, pericardial effusion, arrhythmias, and coronary artery disease of unknown origin. Esophageal involvement was defined by the presence of gastroesophageal reflux, hypomotility on esophageal manometry, or abnormalities on esophageal/intestinal barium radiography.^25^ Gastric symptoms included delayed gastric emptying, bloating, early satiety, nausea, and vomiting. Intestinal symptoms were defined as abdominal distension, chronic diarrhea, fecal incontinence, fecal soilage, constipation, or malabsorption. The EUSTAR disease activity index was calculated using the established composite score incorporating the modified Rodnan skin score (mRSS), tendon friction rubs, elevated C-reactive protein, reduced diffusion capacity of the lung for carbon oxide, and active digital ulcers (DU).^26^ The Charlson Comorbidity Index (CCI) was used to assess the burden of comorbidities through a weighted scoring system covering conditions such as cardiovascular disease, malignancies, and diabetes.^23^

### Follow-up time

In the survival analysis, patients were followed from the enrollment in the SPRING-SIR Registry. Follow-up ended at the earliest occurrence among the date of death, last available clinical assessment, study end date (March 29, 2021), or withdrawal from the registry for any reason.

### Statistical analysis

The data were analyzed using descriptive statistics to calculate the absolute (*n*) and relative (%) frequencies of categorical variables. Continuous variables were described in terms of mean and standard deviation or median and interquartile range, as appropriate following their distribution. All group comparisons were performed using Chi-square test (with Fisher’s correction where applicable), otherwise with ANOVA test. A significance level of 0.05 was used for all tests. Variables with missing data were analyzed using a complete case approach; no data imputation was performed. Sensitivity analyses and multiple imputation were considered; however, given the retrospective nature of the registry, the level and pattern of missingness, and the exploratory aim of the study, we opted for a complete-case analysis. All eligible patients from the SPRING-SIR registry were included in the analysis to ensure representativeness and generalizability of the results.

We used logistic, linear, and multinomial regression models to assess the association between selected predictors (age-RP, age-NRP, and RP-NRP onset pattern) and dichotomous, continuous, and categorical outcomes, respectively.

For all analyses involving the RP-NRP pattern variable, the RP group was used as the reference category, against which the simultaneous and NRP groups were compared. Similarly, when stratifying by age quartiles, the youngest quartile served as the reference.

Kaplan–Meier survival curves and multivariable Cox proportional hazards models were used to investigate the association of the aforementioned predictors with overall survival. Proportional hazards assumptions were tested and multicollinearity between covariates was excluded (*r* < |0.7).

All models were adjusted for potential confounders selected based on prior literature and expert clinical judgment. These included age, sex, cutaneous subset, presence of ILD, CCI.^13,23^

## Results

**Figure 1.**
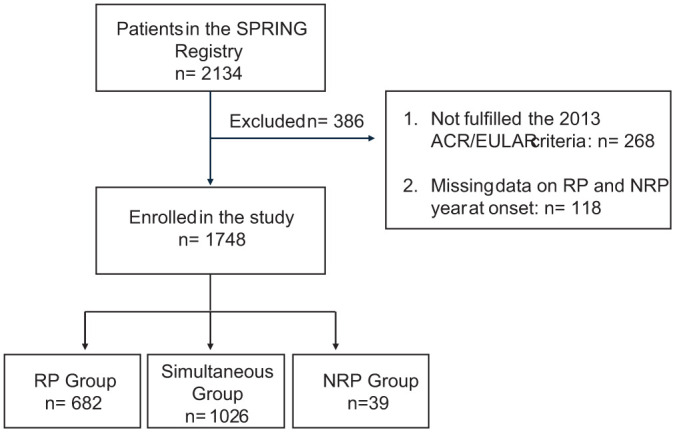
Patients selection process.

This study included a total of 1748/2134 SSc patients from the SPRING-SIR registry (Figure 1); of those, 1549 (86.6%) were females, with a mean age of 59 ± 14 years. Additional clinical characteristics are presented in Table 1.

**Table 1.** General characteristics of the whole population and then stratified according to RP-NRP onset groups.

Clinical characteristics	Total, *n* (%)	Missing, *n* (%)	NRP onset	Simultaneous onset	RP onset	*p*-Value
Patients, *n* (%)	1748 (100)	0	39 (2.2)	1026 (58.8)	682 (39)	
Smoking habit, *n* (%)	530 (33.7)	175 (10)	12 (34.3)	322 (34.5)	196 (32.4)	0.689
Male sex, *n* (%)	199 (11.4)	4 (0.2)	8 (20.5)	133 (13)	58 (8.5)	**0.004**
Age years mean, y, mean ± SD	59 ± 14	1 (0.06)	60 ± 11	58 ± 13	59 ± 14	0.527
Age at RP onset, y, mean ± SD	45 ± 16	1 (0.06)	53 ± 12	49 ± 15	39 ± 15	**<0.001**
Age at NRP onset, y, mean ± SD	48 ± 14	1 (0.06)	48 ± 12	48 ± 15	49 ± 14	0.892
CCI (mean ± SD)	3 ± 2	0	3 ± 2	3 ± 2	3 ± 2	0.276
RP-NRP time, y, mean ± SD	3.94 ± 7.9	0	−4.74 ± 3.44	0.22 ± 0.46	10 ± 9.8	**<0.001**
Skin and vascular						
dcSSc, *n* (%)	351 (20.1)	0	2 (5.1)	259 (25.2)	90 (13.2)	**<0.001**
mRSS, median (IQR)	4 (2–9)	149 (8.5)	3 (0–6)	5 (2–10)	4 (1–8)	**<0.001**
Puffy fingers, *n* (%)	897 (51.9)	20 (1.1)	15 (40.5)	516 (50.8)	366 (54.1)	0.158
Digital ulcers, *n* (%)	368 (21.3)	17 (1)	3 (7.9)	220 (21.7)	145 (21.4)	0.113
Digital pitting scars, *n* (%)	830 (48.1)	21 (1.2)	16 (43.2)	506 (49.9)	308 (45.6)	0.179
Sclerodactyly, *n* (%)	1218 (70.4)	19 (1.1)	28 (73.7)	746 (73.6)	444 (65.6)	**0.002**
Telangiectasias, *n* (%)	1052 (60.8)	18 (1)	25 (65.8)	580 (57.1)	447 (66.1)	**<0.001**
Capillaroscopy pattern late, *n* (%)	409 (26)	176 (10.1)	6 (15.8)	253 (27.7)	150 (24.2)	0.109
Gastrointestinal						
Esophageal symptoms, *n* (%)	885 (51.2)	19 (1.1)	14 (37.8)	550 (54.1)	321 (47.5)	**0.007**
Gastric symptoms, *n* (%)	340 (19.7)	21 (1.2)	5 (13.5)	212 (20.9)	123 (18.2)	0.258
Intestinal symptoms, *n* (%)	350 (20.3)	20 (1.1)	2 (5.4)	205 (20.2)	143 (21.2)	**0.048**
Cardiopulmonary						
Cardiopulmonary manifestation, *n* (%)	487 (28.2)	22 (1.3)	17 (45.9)	314 (31)	156 (23.1)	**<0.001**
Dyspnea, *n* (%)	676 (39.3)	26 (1.5)	18 (48.6)	421 (41.6)	237 (35.3)	**0.018**
ILD, *n* (%)	685 (39.2)	0	16 (41)	428 (41.7)	241 (35.3)	**0.030**
PAH, *n* (%)	28 (3.9)	0	3 (15.8)	10 (2.3)	15 (5.7)	**0.003**
Conduction block, *n* (%)	126 (12.2)	716 (41)	3 (16.7)	87 (14.2)	36 (9)	**0.027**
Pericardial effusion, *n* (%)	100 (7)	319 (18.2)	2 (6.7)	63 (7.4)	35 (6.4)	0.761
Abnormal diastolic function, *n* (%)	331 (23.5)	339 (19.4)	9 (31)	187 (22.3)	135 (25)	0.316
EF (%)	61 ± 6	383 (21.9)	60 ± 9	61 ± 5	61 ± 5	0.652
sPAP (mmHg)	22 ± 16	321 (18.4)	29 ± 19	22 ± 16	23 ± 16	**0.012**
FVC (%)	101 ± 23	440(25.2)	97 ± 24	100 ± 22	104 ± 23	**0.004**
DLCO (%)	69 ± 21	503 (28.8)	65 ± 24	68 ± 20	69 ± 20	0.322
TLC (%)	97 ± 20	990 (56.7)	89 ± 16	96 ± 21	99 ± 20	**0.025**
Musculoskeletal						
Calcinosis, *n* (%)	201 (11.6)	21 (1.2)	5 (13.5)	120 (11.8)	76 (11.3)	0.883
Arthritis, *n* (%)	199 (11.6)	30 (1.7)	3 (8.1)	123 (12.2)	73 (10.9)	0.662
Other symptoms						
Renal crisis, *n* (%)	21 (1.2)	20 (1.1)	1 (2.7)	14 (1.4)	6 (0.9)	0.293
Autoantibodies						
ATA, *n* (%)	597 (34.9)	37 (2.1)	8 (21.6)	411 (41.1)	178 (26.4)	**<0.001**
ARA, *n* (%)	27 (2)	374 (21.4)	0	24 (2.9)	3 (0.6)	**0.007**
ACA, *n* (%)	508 (31.6)	141 (8.1)	9 (26.5)	239 (25.2)	260 (41.5)	**<0.001**

Bold indicates statistically significant result.

ACA, anti-centromere antibodies; ARA, anti-RNA polymerase III antibodies; ATA, anti-topoisomerase antibodies; BMI, body mass index; CCI, Charlson comorbidity index; dcSSc, diffuse cutaneous systemic sclerosis; DLCO, diffusing capacity of the lung for carbon monoxide; EF, ejection fraction; FVC, forced vital capacity; ILD, interstitial lung disease; IQR, interquartile range; mRSS, modified Rodnan skin score; *n*, numbers; NRP, non-Raynaud’s phenomenon; PAH, pulmonary arterial hypertension; RP, Raynaud’s phenomenon; SD, standard deviation; sPAP, systolic pulmonary artery pressure; TLC, total lung capacity; y, years.

### Population characteristics according to the RP-NRP patterns of onset

The RP group consisted of 682 patients (39%) with a mean age-RP of 39 ± 15 years and age-NRP of 49 ± 14 years. The simultaneous group included 1026 (58.8%) patients, in whom age-RP was 49 ± 15 years and age-NRP was 48 ± 15 years. Lastly, the NRP group comprised 39 patients (2.2%), with a mean age-RP of 53 ± 12 years and age-NRP of 48 ± 12 years.

When comparing the three groups, we observed specific differences in the distribution of SSc features (Table 1).

Patients in the RP group were characterized by a higher prevalence of anti-centromere antibodies (ACA, 41.5% vs 25.2% in the simultaneous group and 26.5% in NRP; *p* < 0.001) and earlier age at RP onset (*p* < 0.001). In line with this autoantibody profile, patients in the RP group presented a lower prevalence of ILD (35.3% vs 41.7% in simultaneous group and 41.0% in NRP; *p* = 0.03), dyspnea (35.3% vs 41.7% in simultaneous group and 41.0% in NRP, *p* = 0.018), and sclerodactyly (65.6% vs 73.6% in simultaneous group and 73.7% in NRP, *p* = 0.002), while intestinal symptoms were slightly more common (21.2% vs 20.2% in simultaneous group and 5.4% in NRP, *p* = 0.048).

Patients with simultaneous RP-NRP onset had a higher prevalence of ATA (41.1% vs 26.4% in RP and 21.6% in NRP, *p* < 0.001). Consistently, they presented more frequently with dcSSc (25.2% vs 13.2% in RP and 5.2% in NRP, *p* < 0.001), a higher median mRSS, and more prevalent clinical manifestation such as esophageal involvement (54.1% vs 47.5 in RP and 37.8% in NRP, *p* = 0.007) and ILD (41.7% vs 35.3 in RP and 41% in NRP, *p* = 0.03).

Conversely, NRP patients showed no relevant positivity of a specific autoantibody pattern. Clinically, NRP patients had lower prevalence of dcSSc and esophageal symptoms, but a higher prevalence of cardiopulmonary involvement, specifically of PAH. Consistently, we observed a higher systolic pulmonary artery pressure (sPAP) on echocardiography, lower forced vital capacity (FVC), and total lung capacity, although the latter two fell close to the normal range.

### Population characteristics according to the age of RP and the age of NRP

When dividing our population according to the quartiles of the distribution of age-RP, the first group included 436 patients whose RP appeared before 34 years of age, the second group consisted of 422 patients with RP onset between 35 and 45 years, the third group had 439 patients with RP onset between 46 and 56 years, while the fourth group included patients in whom RP appeared after aging 56 years (450 patients).

The most striking differences were observed when comparing the first and the fourth groups. Patients with RP onset in younger age had a higher prevalence of ATA (42.2% vs 31.5, *p* = 0.003) and consistently showed a greater prevalence of dcSSc (23.2% vs 14.9%, *p* = 0.01), DU (29.2% vs 14.6%, *p* < 0.001), digital pitting scars (58.2% vs 35.7%, *p* < 0.001), and joint contractures (17.1% vs 9.7%, *p* = 0.014). Conversely, the group with RP onset after 56 years of age was characterized by a higher prevalence of cardiopulmonary manifestations (35.3% vs 21.1%, *p* < 0.001) and a numerical increase in sPAP values (25 ± 18 vs 21 ± 14 mmHg, *p* < 0.001). Other features are shown in Table S1.

When clustering our population according to the distribution of the age-NRP, the first group consisted of 311 (17.8%) patients whose NRP symptoms appeared before the age of 38. The second group included 663 (37.9%) patients with NRP onset between 39 and 49 years. The third group comprised 407 (23.3%) patients with NRP onset between 50 and 59 years, while the fourth group, with a total of 367 (21%) patients, included those with NRP sign on symptom onset after the age of 59.

Similarly, when comparing the groups, the most relevant differences were observed between the first and fourth groups. Again, we observed a higher prevalence of ATA (50.7% vs 29.9%, *p* < 0.001), dcSSc (30.2% vs 13.1%, *p* < 0.001), and DU (34.4% vs 14%, *p* < 0.001) in the group with NRP onset at younger age. In contrast, the group with age-NRP over 59 years showed higher prevalence of ACA (35.7% vs 22.8%, *p* = 0.004) and more prominent cardiopulmonary manifestations (36.5% vs 22.4%, *p* < 0.001). Other features are shown in Table S2.

### Associating age-RP, age-NRP, and RP-NRP timing with SSc presentation

Logistic regression analysis revealed that the group with simultaneous RP-NRP onset was associated with a higher risk of presenting the dcSSc subset, compared to the RP group (odds ratio (OR), 1.491, 95% confidence interval (CI): 1.032–2.154, *p* = 0.033), independently of ATA positivity and sex (Table 2). Additionally, we identified age at RP onset as a predictor of sPAP values (β −0.149, 95% CI: −0.297 to −0.001; *p* = 0.049, *N* = 1426) and mRSS (β −0.53, 95% CI: 0.094–0.011; *p* = 0.012, *N* = 1598; Table S3). No statistically significant association was found when predicting other SSc-related manifestations.

**Table 2.** Logistic multivariable regression models predicting clinical features of SSc.

Outcome	*N* patients included	Predictors	OR	95% CI *lower bound*	95% CI *upper bound*	*p*-Value
ILD		RP onset	Reference			0.892
	1705/1748	NRP onset	1.033	0.486	2.197	0.933
	(97.5%)	Simultaneous onset	0.944	0.719	1.240	0.678
		Age_RP	1.014	0.996	1.033	0.119
		Constant	0.093			<**0.001**
dcSSc		RP onset	Reference			0.006
	1705/1748	NRP onset	0.263	0.057	1.206	0.086
	(97.5%)	Simultaneous onset	1.491	1.032	2.154	**0.033**
		Age_RP	1.030	0.997	1.063	0.072
		Constant	0.373			**0.002**
DU		RP onset	Reference			0.218
	1557/1748	NRP onset	0.352	0.099	1.250	0.106
	(89.1%)	Simultaneous onset	1.041	0.740	1.465	0.816
		Age_RP	0.995	0.974	1.018	0.686
		Constant	0.243			<**0.001**
DPS		RP onset	Reference			0.748
	1553/1748	NRP onset	0.907	0.419	1.961	0.804
	(88.8%)	Simultaneous onset	1.091	0.820	1.452	0.550
		Age_RP	0.992	0.974	1.009	0.345
		Constant	0.730			0.266
Calcinosis		RP onset	Reference			0.948
	1701/1748	NRP onset	1.197	0.406	3.534	0.744
	(97.3%)	Simultaneous onset	1.019	0.677	1.534	0.927
		Age_RP	1.003	0.976	1.030	0.846
		Constant	0.098			<0.001
Esophageal symptoms		RP onset	Reference			0.133
	1431/1748	NRP onset	0.653	0.278	1.535	0.329
	(81.9%)	Simultaneous onset	1.215	0.920	1.605	0.170
		Age_RP	1.030	0.997	1.063	0.072
		Constant	0.630			0.238
Gastric symptoms		RP onset	Reference			0.532
	1415/1748	NRP onset	0.484	0.134	1.742	0.267
	(80.9%)	Simultaneous onset	0.921	0.644	1.318	0.654
		Age_RP	1.017	0.992	1.042	0.188
		Constant	0.082			<0.001
Intestinal symptoms		RP onset	Reference			0.171
	1416/1748	NRP onset	0.186	0.024	1.431	0.106
	(81.0%)	Simultaneous onset	1.132	0.802	1.599	0.480
		Age_RP	0.982	0.963	1.002	0.082
		Constant	0.173			<0.001
Diastolic dysfunction		RP onset	Reference			0.450
	1165/1748	NRP onset	1.232	0.450	3.368	0.685
	(66.6%)	Simultaneous onset	0.827	0.570	1.200	0.316
		Age_RP	0.997	0.976	1.019	0.805
		Constant	0.008			<0.001

Mode of onset and age at RP onset represented the predictors of interest. All analyses were adjusted for covariates sex autoantibodies and disease duration.

Bold indicates statistically significant result.

CI, confidence interval; dcSSc, diffuse cutaneous systemic sclerosis; DPS, digital pitting scars; DU, digital ulcers; ILD, interstitial lung disease; NRP, non-Raynaud’s phenomenon; OR, odds ratio; RP, Raynaud’s phenomenon; SSc, systemic sclerosis.

### Predicting the pattern of SSc onset

A multinomial regression analysis was performed to test which characteristics were associated with the risk of belonging to a specific group of RP-NRP onset (Table 3). In this analysis including 1710/1748 (97.8%) patients, we observed that female sex was associated with a higher probability of being included in the RP group (OR, 2.445; 95% CI: 1.052–5.681; *p* = 0.038). Older age at RP onset increased the likelihood of being included in the NRP group (OR, 1.067; 95% CI: 1.042–1.092; *p* < 0.001) or in the simultaneous group (OR, 1.048; 95% CI: 1.041–1.056; *p* < 0.001). Additionally, ACA positivity was associated with a higher probability of being in the RP group (OR, 2.004; 95% CI: 1.550–2.597; *p* < 0.001), while the presence of ATA and anti-RNA polymerase III (ARA) antibodies was linked to a higher likelihood of being included in the Simultaneous group (OR, 1.671; 95% CI: 1.295–2.157; *p* < 0.001 and OR, 4.002; 95% CI: 1.151–13.914; *p* = 0.029, respectively).

**Table 3.** Multinomial logistic regression predicting NRP and Simultaneous onset patterns compared to RP onset in patients with systemic sclerosis.

Group	Predictor	OR	95% CI lower bound	95% CI upper bound	*p*-value
RP ONSET	Reference				
NRP ONSET	Intercept				<**0.001**
	Age_RP	1.067	1.042	1.092	<**0.001**
	Female sex	0.409	0.176	0.950	**0.038**
	ACA	0.329	0.147	0.738	**0.007**
	ATA	0.511	0.212	1.230	0.134
	ARA	2.060E−8	2.060E−8	2.060E−8	NA
SIMULTANEOUS ONSET	Intercept				<**0.001**
	Age_RP	1.048	1.041	1.056	<**0.001**
	Female sex	0.726	0.513	1.027	0.071
	ACA	0.499	0.385	0.645	<**0.001**
	ATA	1.671	1.295	2.157	<**0.001**
	ARA	4.002	1.151	13.914	**0.029**

The analyses included age at RP onset, sex, autoantibodies as predictors. Bold indicates statistically significant result.

ACA, anti-centromere antibodies; ARA, anti-RNA polymerase III antibodies; ATA, anti-topoisomerase I antibodies; CI, confidence interval; NA: not applicable; NRP, non-Raynaud’s phenomenon; OR, odds ratio; RP, Raynaud’s phenomenon.

### Survival analysis

Survival status was available for 943 patients (53.95%), with median follow-up duration of 24 (interquartile range: 12–48) months and 44 (4.7%) deaths recorded.

**Figure 2.**
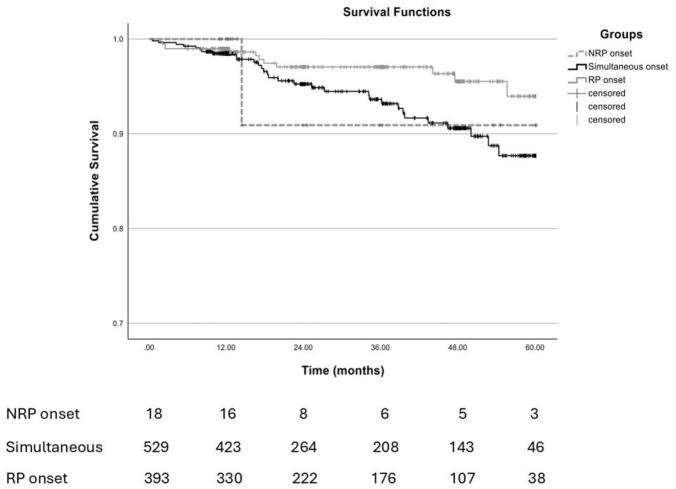
Kaplan–Meier curves illustrating cumulative survival by disease onset pattern. Patients with RP-first onset show better survival compared to those with simultaneous or NRP-first onset. Tick marks indicate censored observations. Differences were assessed using the log-rank test. NRP, non-Raynaud’s phenomenon onset; RP, Raynaud’s phenomenon onset; SG, Simultaneous group onset.

The estimated survival rates were comparable among the RP (97%), NRP (95%), and simultaneous (94%) groups (*p* = 0.09), as presented in Figure 2.

**Figure 3.**
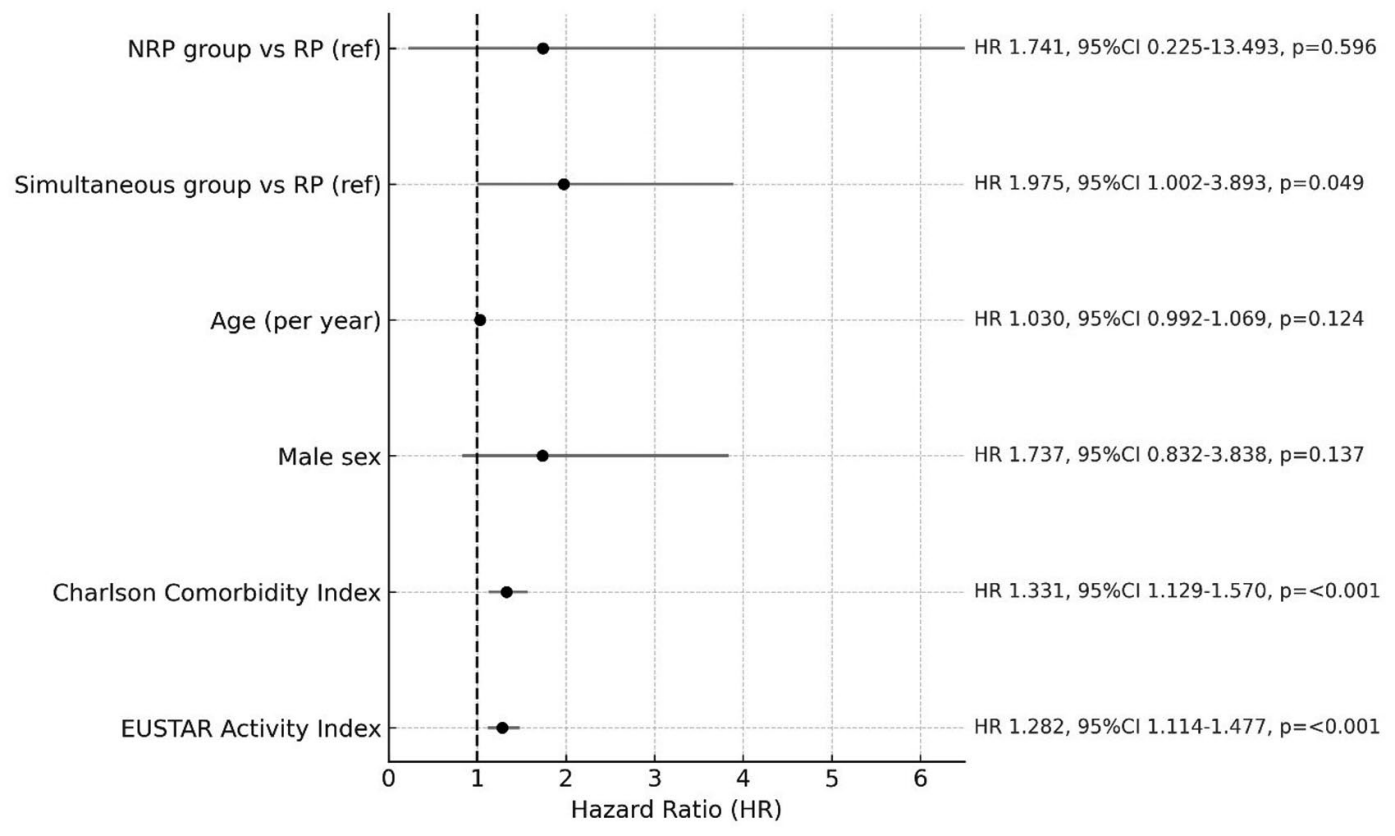
Prognostic factors related to mortality in a Cox regression model. EUSTAR, European Scleroderma Trial and Research; NRP, non-Raynaud’s phenomenon; RP, Raynaud’s phenomenon.

However, when testing the risk carried by the RP-NRP onset patterns in an exploratory, multivariable Cox regression model, including 936/943 (99.3%) of the patients with follow-up data, we observed that the simultaneous group had a significant higher risk of mortality compared to RP group (hazard ratio (HR), 1.975; 95% CI: 1.002–3.893, *p* = 0.049), which was independent from age at enrollment in the registry, sex, EUSTAR disease activity index, and CCI (Figure 3).

As expected, Kaplan–Meier survival analysis revealed lower survival rates for patients with older age at time of RP and NRP onset, respectively, 94% and 95.2% and significantly lower than younger ages on onset (data not shown).

## Discussion

Our study provides a comprehensive analysis of SSc patients enrolled in the Italian SPRING-SIR registry, emphasizing the influence of the pattern of RP-NRP timing, the age of RP onset, and the age of the first NRP sign or symptom on the clinical presentation of SSc, as well as preliminary results on a possible impact on outcomes.

The mode of clinical onset in SSc remains a critical area of investigation, particularly regarding the differences between RP onset and NRP onset. Although RP is the expected initial manifestation in most SSc patients, studies have highlighted the distinct and aggressive nature of NRP onset.^6,27^ These studies confirm and expand on previous observations from the SSc Study Group of the Italian Society of Rheumatology, which reported a significant correlation between the mode of disease onset (RP or NRP) and adverse SSc features, including the dcSSc subset, ATA seropositivity, and reduced 10-year survival.^10,11^ More recently, another Italian study examined the sine scleroderma subset in the SPRING registry, showing a longer RP-to-diagnosis interval in ssSSc than in lcSSc and dcSSc.^12^ Comparably, two analysis of the international EUSTAR registry are in line with our data. While Hügle et al.^28^ demonstrated that late-onset SSc was associated with a higher prevalence of pulmonary hypertension and cardiac dysfunction, but milder cutaneous involvement and worse survival, Jaeger et al.^29^ confirmed that age and timing of symptom onset influenced the risk and time of onset of major organ manifestations, including ILD and PAH. These observations are consistent with our results and support the face validity of our patient stratification system based on mode of onset. Unlike these earlier works, our study broadens the framework by introducing a distinct “Simultaneous onset” group, integrated age at RP and NRP onset, explored its relationship with antibody profiles and clinical phenotypes, and performed survival analyses adjusted for comorbidities. These aspects extend previous findings and highlight the potential of onset patterns as a practical tool for patient stratification in real-life.

The Spanish Scleroderma Registry reported that approximately 17% of patients presented with NRP onset, which was associated with a higher likelihood of developing dcSSc and an increased risk of severe outcomes, including early visceral involvement, ILD, and PAH. Interestingly, our cohort revealed a markedly lower proportion of patients with NRP-first onset (2.2%). This discrepancy may reflect differences in study design, patient selection, or data collection methods, particularly regarding the timing and characterization of early symptoms. Notably, the inclusion in our study of a distinct “Simultaneous onset” group, representing patients who developed RP and NRP symptoms within the same year, may have contributed to a more refined classification. This intermediate category potentially encompassed individuals who, in other cohorts, might have been classified under the NRP group, thereby explaining the lower prevalence observed in our data.

By distinguishing the simultaneous group, our classification captures the heterogeneity of SSc onset more effectively and identifies a subset of patients with a particularly aggressive clinical course. This approach strengthens early risk stratification and supports tailored management strategies aimed at improving outcomes. Interestingly, a similar approach has been applied in other connective tissue diseases. In antisynthetase syndrome (aSyS), the temporal sequence of clinical features at onset has been linked to specific phenotypes and outcomes. In large international cohorts, earlier onset of aSyS-ILD was associated with more severe disease courses and worse pulmonary outcomes.^30,31^ Although the pathogenetic mechanisms and target organs differ from SSc, these findings reinforce the notion that the timing of symptom onset is a clinically relevant stratification tool across autoimmune connective tissue diseases, supporting our approach to clustering patients into groups with different mode of onset.

While NRP onset has been associated with the presence of dcSSc,^32^ evidence regarding its direct impact on survival remains limited. The absence of robust survival studies hampers the ability to fully evaluate the prognostic significance of NRP onset and its role in long-term outcomes.

In contrast, more is known about age-related differences in RP onset. Indeed, older age at onset is associated with ACA positivity and lcSSc, while younger onset more commonly linked to dcSSc and ATA antibodies.^28,33–35^ However, late-onset patients also face worse outcomes, including higher rates of organ involvement such as ILD, PAH, and cardiac complications.^36^

In the present study, the division of patients into the RP, simultaneous, and NRP onset subgroups identify more clearly the different clinical–prognostic SSc scenarios observable in real life because of the different timing of RP onset.

It allows us to reveal significant differences in the distribution of clinical manifestations and autoantibody profiles, supporting the role of RP-NRP onset pattern in the disease stratification. The RP group was characterized by a younger mean age at RP onset and a higher prevalence of ACA, frequently associated with a milder clinical endotype, including a lower frequency of dcSSc, ILD, and dyspnea. These findings are consistent with previous observations that lcSSc is often associated with a longer interval between the onset of RP and the appearance of the first NRP sign or symptom.^11,32^

The patients in the simultaneous group represented the largest part of our cohort (58.8%) and presented a more severe disease endotype, characterized by a higher prevalence of ATA, dcSSc, and esophageal involvement.

In contrast, the NRP group demonstrated a higher prevalence of cardiopulmonary complications, including higher sPAP, a higher rate of PAH, and lower FVC values. These findings suggest that NRP onset may identify a subgroup of patients at increased risk for severe organ complications. This highlights the crucial role of non-rheumatologists who often are the first healthcare contact for these patients, making their role in identifying early signs of SSc. Raising awareness of these clinical patterns among primary care providers and other specialists might indeed improve early diagnosis and timely referral.

Moreover, our data revealed that the age at onset of RP and NRP symptoms may also significantly influence the disease endotype. Consistent with previous studies,^28^ we confirmed that onset of both RP and NRP in younger ages were associated with higher prevalence of ATA positivity, severe skin, and vascular involvement. Conversely, older RP onset and older NRP onset (respectively, >56 and >59 years in our cohort), were characterized by milder cutaneous features but associated with more severe cardiopulmonary involvement and increased prevalence of PAH.^37^ Similar data were showed by the EULAR Scleroderma Trials and Research (EUSTAR) group in a previous work in which they demonstrated that late-onset SSc was an independent risk factor for pulmonary hypertension.^28^ The demographic and clinical findings from the Italian cohort described by Ferri et al. also underscore these age- and endotype-related outcomes. In their study of 1012 Italian SSc patients, significant differences in survival rates and organ involvement were observed based on cutaneous subset and age of onset.^10^

Other studies identified an older age at the time of SSc diagnosis being linked to poorer outcomes, including an increased risk of mortality,^38–41^ mainly due to a more aggressive disease course,^42^ as well as to the impact of biological age itself. Specifically, some authors identified that the risk of death increases by 5% for every 1-year increase in age at time of diagnosis.^3^ Age-related survival and disease characteristics further highlighted that both older RP and NRP onset groups had lower 5-year survival rates. For older patients, our findings align with other cohort studies showing a higher prevalence of cardiac complications such as conduction blocks, left ventricular diastolic dysfunction, and increased sPAP.^28,33,34,39,43–45^ which remained significant after adjustment for sex, antibody status, and SSc subtype.

Our study additionally demonstrated that the clinical onset of SSc in terms of RP-NRP patterns of presentation may represent an additional independent risk factor for mortality in SSc, in line with the data previously reported by the Spanish Scleroderma Study Group.^27^ Although the survival curves showed comparable mortality rates across the RP, simultaneous, and NRP groups, we observed a significantly higher mortality risk in the simultaneous group compared to the RP group after adjustment for demographic and disease characteristics acting as confounders. This finding further underlines the association between a short-term, simultaneous RP-NRP timing in the onset of SSc and a more aggressive disease endotype, which adds up to the previously known predictors of mortality.

**Figure 4.**
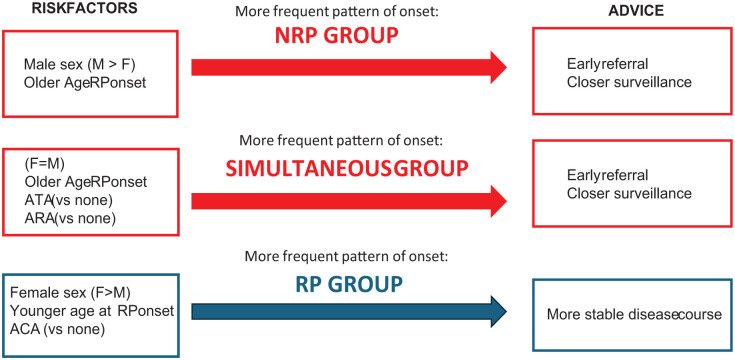
Classification of onset groups (NRP, Simultaneous, RP) based on risk factors, predominant clinical characteristics, and management recommendations. Risk factors include sex, age at RP onset, and the presence of autoantibodies (ATA, ARA, ACA). The arrows indicate the most frequent patterns of onset, with corresponding advice for each group highlighted. ACA, anti-centromere antibodies; ARA, anti-RNA polymerase III antibodies; ATA, anti-topoisomerase I antibodies; NRP, non-Raynaud’s phenomenon; RP, Raynaud’s phenomenon.

Given the impact that the RP-NRP pattern of onset carries on both disease presentation and prognosis, we tried to identify which features were associated with each specific group, specifically focusing on demographic and autoantibody data. Indeed, based on the results of our multinomial regression models, clinicians may apply these predictive factors to assist in the early identification of patients who may benefit from earlier referral to an SSc specialist center for further evaluation and management. In fact, patients exhibiting characteristics associated with a higher likelihood of belonging to the RP group (e.g., female sex, ACA positivity, early RP onset) may have a more stable disease course. In contrast, patients who are more likely to fall into the NRP or simultaneous groups (e.g., older age of RP onset, male sex, ATA, or ARA positivity) should be prioritized for early referral and closer surveillance to mitigate the risk of severe disease progression (see Figure 4).

Our study benefits from the large sample size and comprehensive data from a national registry, allowing robust subgroup analysis and supporting the potential clinical implications of our results. In terms of risk stratification and personalized management, the timing of RP onset and age of first NRP symptoms may indeed guide risk assessment, helping clinicians identify patients at greater risk for severe disease or mortality. Importantly, onset timing could also serve as a simple, readily available marker to be incorporated into future risk stratification tools or even contribute to refinement of classification criteria, complementing the current emphasis on RP as an entry symptom. In particular, the identification of simultaneous onset at diagnosis could be used as an early “red flag” to prioritize closer monitoring and timely therapeutic intervention (e.g., HRCT, PFT, echocardiography), and timely therapeutic intervention. Similarly, NRP-first onset may identify patients requiring an analogous intensive approach. Conversely, RP-first onset at a younger age may indicate a more indolent course, allowing for a less intensive but still regular follow-up. Beyond individual management, onset timing and age could be integrated into simple clinical algorithms or future prediction models to stratify patients at diagnosis, optimize use of healthcare resources, and inform tailored treatment strategies. This information could also support patient counseling by providing early prognostic insights and may help stratify subgroups in clinical trials, facilitating the design of studies focused on high-risk patients. Further validation in international cohorts will be essential to determine whether these patterns can be robustly translated into practical decision-making tools and incorporated into long-term management pathways.

## Limitations

Notwithstanding, several limitations must be acknowledged in our analysis. First, the cross-sectional design of our study does not allow conclusions on causality and limits the ability to evaluate disease progression over time. Longitudinal data analysis is therefore necessary to confirm and refine these associations, as well as to support the potential clinical implications. Second, the retrospective nature of the study and the use of complete cases without missing data for all inferential and regression analysis may contribute to a potential selection bias. A further limitation of our study is that no a priori power analysis was performed to calculate the sample size, as this was a retrospective, post hoc, registry-based analysis of prospectively collected data.

Third, only about half of the cohort had available survival data, and the median follow-up of 24 months limits the ability to capture late events, possibly introducing informative censoring and bias in survival estimates. In addition, treatment exposure was not included in regression or survival models because of incomplete longitudinal data, which may have influenced organ outcomes and mortality estimates. This limitation is further compounded by small numbers of deaths recorded and the lack of cause-specific mortality data in the SPRING registry, which reduces the precision of our survival analyses.

Moreover, the age at RP onset was patient-reported and therefore subject to recall bias especially when RP preceded SSc diagnosis by several years. This limitation may also affect the simultaneous group, in which we were unable to ascertain whether RP or NRP occurred first within the same year. This may introduce uncertainty in classification, suggesting that some cases might have been misclassified within this category. Additionally, the lack of detailed information for RP and NRP onset date may have improperly assigned certain patients to the RP or NRP groups, given their onset being recorded in two consecutive calendar years, while still potentially within 12 months from each other.

In addition, the limited size of the NRP group (corresponding to 2.2% of the overall population) and the in our median short-term observation may limit the generalizability of our findings.

Finally, longitudinal analyses in larger international cohorts, overcoming the abovementioned limitations are planned to support our results and confirm whether the prognostic impact of RP-NRP onset timing persists over extended periods, particularly in the NRP group.

## Conclusion

In conclusion, our study highlights the importance of stratifying SSc patients not only using cutaneous subset and autoantibody patterns but also based on the age of RP/NRP onset and the RP/NRP pattern of presentation. We identified significant heterogeneity in disease manifestations and outcomes across different patterns of onset, further supporting the need for personalized management approaches in SSc, with a focus on early identification and risk stratification to optimize clinical outcomes. Further longitudinal studies are required to confirm these associations and refine strategies for early intervention and monitoring.

## Supplemental Material

sj-docx-1-tab-10.1177_1759720X251410243 – Supplemental material for Age and onset timing of Raynaud’s phenomenon and first non-Raynaud symptom as prognostic factors in systemic sclerosis: a retrospective analysis from the Italian national multicenter Systemic Sclerosis Progression INvestiGation registry of the Italian Society for Rheumatology (SPRING-SIR) Supplemental material, sj-docx-1-tab-10.1177_1759720X251410243 for Age and onset timing of Raynaud’s phenomenon and first non-Raynaud symptom as prognostic factors in systemic sclerosis: a retrospective analysis from the Italian national multicenter Systemic Sclerosis Progression INvestiGation registry of the Italian Society for Rheumatology (SPRING-SIR) by Silvia Peretti, Cosimo Bruni, Francesco Bonomi, Rossella De Angelis, Gianluigi Bajocchi, Dilia Giuggioli, Martina Orlandi, Giovanni Zanframundo, Roberta Foti, Elisa Visalli, Giovanna Cuomo, Alarico Ariani, Edoardo Rosato, Gemma Lepri, Francesco Girelli, Valeria Riccieri, Elisabetta Zanatta, Silvia Laura Bosello, Ilaria Cavazzana, Francesca Ingegnoli, Maria De Santis, Fabio Cacciapaglia, Giuseppe Murdaca, Giuseppina Abignano, Giorgio Pettiti, Alessandra Della Rossa, Maurizio Caminiti, Annamaria Iuliano, Giovanni Ciano, Lorenzo Beretta, Gianluca Bagnato, Ennio Lubrano, Ilenia De Andres, Luca Idolazzi, Marta Saracco, Cecilia Agnes, Corrado Campochiaro, Giacomo De Luca, Edoardo Cipolletta, Marco Fornaro, Federica Lumetti, Amelia Spinella, Luca Magnani, Veronica Codullo, Carlo Iandoli, Antonietta Gigante, Greta Pellegrino, Erika Pigatto, Maria Grazia Lazzaroni, Enrico De Lorenzis, Gianna Angela Mennillo, Marco Di Battista, Giuseppa Pagano Mariano, Federica Furini, Licia Vultaggio, Simone Parisi, Clara Lisa Peroni, Gerolamo Bianchi, Enrico Fusaro, Gian Domenico Sebastiani, Marcello Govoni, Salvatore D’Angelo, Franco Cozzi, Franco Franceschini, Serena Guiducci, Lorenzo Dagna, Andrea Doria, Carlo Salvarani, Maria Antonietta D’Agostino, Florenzo Iannone, Marco Matucci-Cerinic, Clodoveo Ferri and Silvia Bellando Randone in Therapeutic Advances in Musculoskeletal Disease

## Declarations

**Ethics approval and consent to participate:** The study was approved by the coordinating ethics committee of AOU Careggi, Florence, Italy (Ref. OSS 15.010), and by the ethics committees of all participating centers. Written informed consent was obtained from all participants before enrollment in the SPRING-SIR registry.

**Consent for publication:** Not applicable.

**Availability of data and materials:** The data that support the findings of this study were derived from the SPRING-SIR Registry of the Italian Society for Rheumatology. Data from the SPRING-SIR Registry are not publicly available due to institutional and ethical restrictions, but may be accessed upon reasonable request to the Italian Society of Rheumatology and subject to approval by the SPRING-SIR Steering Committee.
