# Supplementary material for: Age and onset timing of Raynaud’s phenomenon and first non-Raynaud symptom as prognostic factors in systemic sclerosis: a retrospective analysis from the Italian national multicenter Systemic Sclerosis Progression INvestiGation registry of the Italian Society for Rheumatology (SPRING-SIR)
Source: Ther Adv Musculoskelet Dis. 2026 Feb 5;18:1759720X251410243. doi: 10.1177/1759720X251410243 (PMC12881321; doi:10.1177/1759720X251410243)
Supplement: sj-docx-1-tab-10.1177_1759720X251410243 – Supplemental material for Age and onset timing of Raynaud’s phenomenon and first non-Raynaud symptom as prognostic factors in systemic sclerosis: a retrospective analysis from the Italian national multicenter Systemic Sclerosis Progression INvestiGation regi [file sj-docx-1-tab-10.1177_1759720X251410243.docx]

SUPPLEMENTAL MATERIAL

|  | TOTAL | MISSING n (%) | ≤ 34 y | 35-45 y | 46-56 y | > 56 y | P-value |
| --- | --- | --- | --- | --- | --- | --- | --- |
| Patients, n (%) | 1747 (99.9) | 1 (0.01) | 436 (24.9) | 422 (24.1) | 439 (25.1) | 450 (25.7) |  |
| Smoking ever, n (%) | 530 (33.7) | 175 (10) | 123 (31.9) | 150 (39.9) | 147 (36.6) | 110 (27) | **<0.001** |
| Male sex, n (%) | 199 (11.4) | 4 (0.2) | 47 (10.8) | 40 (9.5) | 60 (13.7) | 52 (11.6) | 0.272 |
| Age, years, mean ± SD | 59 ± 14 | 1 (0.06) | 46 ±13 | 55 ±11 | 61 ±8 | 71 ± 7 | **<0.001** |
| BMI, mean ± SD | 24.07 ±4.3 | 188 (10.7) | 23.01 ± 4.10 | 24.01 ± 4.30 | 24.67 ± 4.48 | 24.58 ± 4.11 | **<0.001** |
| CCI, mean ± SD | 3±2 | 0 | 2 ± 2 | 2 ± 2 | 3 ±2 | 5 ±2 | **<0.001** |
| mRSS, mean ± SD | 4 (2-9) | 149 (8.5) | 5 (2-11) | 4 (2-9) | 4 (2-9) | 3 (0-6) | **0.007** |
| RP-NRP time, years, mean ± SD | 3.94 ± 7.9 | 0 | 8.77 ± 11.67 | 4.03 ±7.26 | 1.97 ±4.17 | 1.11 ±3.06 | **<0.001** |
| **Skin and vascular** |  | | | | | | |
| dcSSc, n (%) | 351 (20.1) | 0 | 102 (23.4) | 91 (21.6) | 91 (20.7) | 67 (14.9) | **0.010** |
| Puffy fingers, n (%) | 896 (51.9) | 20 (1.1) | 208 (48.3) | 210 (50.2) | 256 (59) | 222 (50%) | **0.007** |
| Digital ulcers, n (%) | 368 (21.3) | 17 (1) | 126 (29.2) | 88 (21.1) | 89 (20.5) | 65 (14.6) | **<0.001** |
| Pitting scars, n (%) | 830 (48.1) | 21 (1.2) | 251 (58.2) | 223 (53.5) | 197 (45.5) | 159 (35.7) | **<0.001** |
| Sclerodactily, n (%) | 1218 (70.5) | 19 (1.1) | 313 (72.5) | 300 (71.8) | 292 (67.4) | 313 (70.3) | 0.381 |
| Teleangectasias, n (%) | 1051 (60.8) | 18 (1) | 275 (63.7) | 261 (62.6) | 259 (59.7) | 256 (57.4) | 0.219 |
| Capillaroscopy pattern late, n (%) | 409 (26) | 176 (10.1) | 116 (30.1) | 97 (25.6) | 105 (26.3) | 91 (22.4) | 0.110 |
| **Gastrointestinal** |  | | | | | | |
| Esophageal involvement, n (%) | 884 (51.2) | 20 (1.1) | 215 (49.8) | 223 (53.3) | 231 (53.3) | 215 (48.3) | 0.330 |
| Gastric involvement, n (%) | 339 (19.6) | 22 (1.2) | 65 (15.1) | 86 (20.6) | 89 (20.6) | 99 (22.2) | **0.044** |
| Intestinal involvement, n (%) | 350 (20.3) | 20 (1.1) | 100 (23.2) | 98 (23.4) | 77 (17.8) | 75 (16.9) | **0.021** |
| **Cardiopulmonary** |  | | | | | | |
| Cardiopulmonary manifestation, n (%) | 487 (28.2) | 22 (1.2) | 91 (21.1) | 115 (27.5) | 125 (28.9) | 156 (35.3) | **<0.001** |
| Dyspnea, n (%) | 676 (39.3) | 26 (1.5) | 128 (29.7) | 164 (39.3) | 190 (44.2) | 194 (43.8) | **< 0.001** |
| ILD, n (%) | 684 (39.2) | 0 | 167 (38.3) | 155 (36.7) | 174 (39.6) | 188 (41.8) | 0.473 |
| PAH, n (%) | 28 (3.9) | 0 | 2 (1.1) | 7 (4.3) | 6 (3.3) | 13 (6.5) | 0.054 |
| Conduction block, n (%) | 125 (12.1) | 716 (41) | 25 (9.5) | 20 (8.8) | 36 (13.3) | 44 (16.2) | **0.037** |
| Pericardial effusion, n (%) | 99 (6.9) | 319 (18.2) | 18 (4.9) | 35 (10.1) | 23 (6.4) | 23 (6.4) | **0.044** |
| Abnormal diastolic function, n (%) | 330 (23.4) | 339 (19.4) | 51 (14.2) | 69 (20.2) | 89 (25.2) | 121 (34) | **<0.001** |
| EF, (%) | 61 ±6 | 383 (21.9) | 62 ± 5 | 62 ±5 | 61 ±6 | 61 ±6 | **0.005** |
| sPAP mmHg, n ± SD | 22 ±16 | 321 (18.4) | 21 ±14 | 21 ±16 | 23 ±16 | 25 ±18 | **<0.001** |
| FVC, (%) ± SD | 101 ±23 | 440(25.2) | 99 ± 21 | 101 ±22 | 102 ± 23 | 104 ± 25 | **0.027** |
| DLCO, (%) ± SD | 69 ±21 | 503 (28.8) | 70 ± 20 | 67 ±21 | 70 ±20 | 67 ±21 | 0.084 |
| **Musculoskeletal** |  | | | | | | |
| Calcinosis, n (%) | 201 (11.6) | 21 (1.2) | 63 (14.6) | 55 (13.2) | 48 (11.1) | 35 (7.9) | **0.013** |
| Arthritis n (%) | 198 (11.5) | 30 (1.7) | 44 (10.3) | 45 (10.8) | 58 (13.5) | 51 (11.5) | 0.472 |
| Joint contractures n (%) | 231 (13.4) | 20 (1.1) | 74 (17.1) | 57 (13.7) | 57 (13.2) | 43 (9.7) | **0.014** |
| **Autoantibodies** |  | | | | | | |
| ANA+, n (%) | 1663 (97.4) | 39 (2.2) | 415 (97) | 398 (96.4) | 419 (98.6) | 431 (97.5) | 0.222 |
| ATA, n (%) | 596 (34.9) | 37 (2.1) | 180 (42.2) | 139 (33.7) | 138 (32.2) | 139 (31.5) | **0.003** |
| ARA, n (%) | 27 (2) | 374 (21.4) | 2 (0.6) | 5 (1.5) | 11 (3.1) | 9 (2.5) | 0.089 |
| ACA, n (%) | 508 (31.6) | 141 (8.1) | 115 (29.3) | 114 (29.5) | 131 (32.3) | 148 (35.2) | 0.232 |
| **Therapies** |  | | | | | | |
| Ongoing bDMARDs, n (%) | 94 (5.4) | 0 | 36 (8.3) | 29 (6.9) | 19 (4.3) | 10 (2.2) | **<0.001** |
| Ongoing csDMARDS, n (%) | 577 (33) | 0 | 135 (31) | 145 (34.4) | 159 (36.2) | 138 (30.7) | 0.228 |
| Ongoing Ccs, n (%) | 631 (36.1) | 0 | 137 (31.4) | 146 (34.6) | 182 (41.5) | 166 (36.9) | **0.017** |
| Ongoing ERA, n (%) | 399 (22.8) | 0 | 126 (28.9) | 108 (25.6) | 95 (21.6) | 70 (15.6) | **<0.001** |
| Ongoing PDE5, n (%) | 79 (4.5) | 0 | 20 (4.6) | 21 (5) | 21 (4.8) | 17 (3.8) | 0.841 |
| Ongoing Prost, n (%) | 995 (57) | 0 | 278(63.8) | 264 (62.6) | 255 (58.1) | 198 (44) | **<0.001** |

Table 1 Supplementary: General characteristics according to age at RP onset

*NRP non-Raynaud’s phenomenon, RP Raynaud’s phenomenon, SD standard deviation, n numbers, y years, IQR Interquartile Range, BMI Body Mass index, CCI Charlson comorbidity index, mRSS modified Rodnan skin score, ILD interstitial lung disease, PAH pulmonary arterial hypertension, EF Ejection Fraction, sPAP Systolic Pulmonary Artery Pressure, FVC Forced Vital Capacity, DLCO Diffusing Capacity of the Lung for Carbon Monoxide, ANA antinuclear antibodies, ATA anti-topisomerase antibodies, ARA Anti-RNA polymerase III antibodies, ACA anti-centromere antibodies, bDMARDs Biologic Disease-Modifying Antirheumatic, csDMARDS Conventional Synthetic Disease-Modifying Antirheumatic Drugs, Ccs Corticosteroids, ERA Endothelin Receptor Antagonists, PDE5 Phosphodiesterase type 5 inhibitors, Prost Prostanoids,*

|  | TOTAL n (%) | MISSING n(%) | ≤ 38 years | 38-49 years | 49-59 years | > 59 years | P-value |
| --- | --- | --- | --- | --- | --- | --- | --- |
| Patients, n (%) | 1748 (100) | 0 | 311 (17.8) | 663 (37.9) | 407 (23.3) | 367 (21.0) |  |
| Smoking habit, n (%) | 530 (33.7) | 175 (10) | 78 (28.6) | 222 (37.4) | 142 (38) | 88 (26.5) | **< 0.001** |
| Male sex, n (%) | 199 (11.4) | 4 (0.2) | 36 (11.6) | 70 (10.6) | 56 (13.8) | 37 (10.1) | 0.352 |
| Age, years, mean ± SD | 59 ± 14 | 1 (0.06) | 42 ± 11 | 56 ±10 | 64 ±7 | 73 ± 6 | **<0.001** |
| BMI, mean ±SD | 24.07 ± 4.3 | 188 (10.7) | 22.64 ±4.09 | 24.19 ±4.44 | 24.34 ±4.22 | 24.79 ±4.06 | **<0.001** |
| CCI, mean ± SD | 3 ± 2 | 0 | 1 ±1 | 2 ±2 | 4 ±2 | 5 ±2 | **<0.001** |
| mRSS, mean ± SD | 4 (2-9) | 149 (8.5) | 6 (2-13) | 4 (2-9) | 4 (2-9) | 3 (0-6) | **<0.001** |
| RP-nonRP time, years, mean ± SD | 3.94 ± 7.9 | 0 | 2.6 ± 4.66 | 5.26 ± 7.74 | 4.55 ±9.92 | 2.02 ±7.25 | **<0.001** |
| Skin and vascular |  |  |  |  |  |  |  |
| dcSSc n (%) | 351 (20.1) | 0 | 94 (30.2) | 130 (19.6) | 79 (19.4) | 48 (13.1) | **<0.001** |
| Puffy fingers, n (%) | 896 (51.9) | 20 (1.1) | 133 (43.2) | 360 (55) | 231 (57.2) | 173 (47.9) | **<0.001** |
| Digital ulcers, n (%) | 368 (21.3) | 17 (1) | 106 (34.4) | 139 (21.2) | 72 (17.8) | 51 (14) | **<0.001** |
| Pitting scars, n (%) | 830 (48.1) | 21 (1.2) | 196 (63.6) | 340 (52) | 168 (41.7) | 126 (34.8) | **<0.001** |
| Sclerodactily, n (%) | 1218 (70.5) | 19 (1.1) | 242 (78.6) | 460 (70.1) | 267 (66.3) | 249 (68.8) | **0.003** |
| Teleangectasias, n (%) | 1051 (60.8) | 18 (1) | 191 (62) | 408 (62.2) | 245 (60.8) | 208 (57.3) | 0.46 |
| Capillaroscopy pattern late, n (%) | 409 (26) | 176 (10.1) | 91 (33.3) | 146 (24.4) | 102 (27.5) | 70 (21.2) | **0.005** |
| Gastrointestinal |  |  |  |  |  |  |  |
| Esophageal involvement, n (%) | 884 (51.2) | 19 (1.1) | 165 (53.6) | 340 (51.9) | 208 (51.5) | 172 (47.5) | 0.423 |
| Gastric involvement, n (%) | 339 (19.6) | 21 (1.2) | 53 (17.3) | 129 (19.7) | 82 (20.3) | 76 (21) | 0.654 |
| Intestinal involvement, n (%) | 350 (20.3) | 20 (1.1) | 67 (21.8) | 148 (22.6) | 73 (18.1) | 62 (17.1) | 0.112 |
| Cardiopulmonary |  |  |  |  |  |  |  |
| Cardiopulmonary manifestation, n (%) | 487 (28.2) | 22 (1.3) | 69 (22.4) | 161 (24.6) | 126 (31.2) | 131 (36.5) | **<0.001** |
| Dyspnea, n (%) | 676 (39.3) | 26 (1.5) | 94 (30.5) | 253 (38.7) | 169 (42.1) | 160 (44.4) | **0.001** |
| ILD, n (%) | 684 (39.2) | 0 | 130 (41.8) | 238 (35.9) | 164 (40.3) | 153 (41.7) | 0.167 |
| PAH, n (%) | 28 (3.9) | 0 | 1 (0.8) | 8 (3.2) | 7 (4) | 12 (7.3) | **0.031** |
| Conduction block, n (%) | 125 (12.1) | 716 (41) | 18 (9.6) | 38 (10.2) | 31 (12.4) | 39 (17.6) | **0.033** |
| Pericardial effusion, n (%) | 99 (6.9) | 319 (18.2) | 13 (5.1) | 41 (7.6) | 29 (8.6) | 17 (5.8) | 0.304 |
| Abnormal diastolic function, n (%) | 330 (23.4 ) | 339 (19.4) | 31 (12.3) | 103 (19.3) | 98 (29.2) | 99 (34.5) | **<0.001** |
| EF, (%) | 61 ±6 | 383 (21.9) | 62 ±5 | 62 ±5 | 61 ±6 | 60 ±6 | **<0.001** |
| sPAP, (mmHg) | 22 ±16 | 321 (18.4) | 20±13 | 21 ±16 | 24 ±16 | 26±18 | **<0.001** |
| FVC, (%) ± SD | 101 ±23 | 440(25.2) | 96 ±20 | 101 ±22 | 102 ±22 | 104 ±25 | **<0.001** |
| DLCO, (%) ± SD | 69 ±21 | 503 (28.8) | 69 ±19 | 69 ±21 | 69 ±21 | 66 ±20 | 0.327 |
| Musculoskeletal |  |  |  |  |  |  |  |
| Calcinosis, n (%) | 201 (11.6) | 21 (1.2) | 55 (17.9) | 78 (11.9) | 41 (10.1) | 27 (7.5) | **<0.001** |
| Arthritis, n (%) | 198 (11.5 ) | 30 (1.7) | 34 (11.2) | 80 (12.3) | 46 (11.4) | 39 (10.8) | 0.901 |
| Joint contractures, n (%) | 231 (13.4) | 20 (1.1) | 60 (19.5) | 87 (13.3) | 50 (12.4) | 34 (9.4) | **0.002** |
| Autoantibodies |  |  |  |  |  |  |  |
| ANA+, n (%) | 1663 (97.4) | 39 (2.2) | 296 (97) | 631 (97.1) | 386 (98) | 351 (97.5) | 0.828 |
| ATA, n (%) | 596 (34.9) | 37 (2.1) | 154 (50.7) | 215 (33) | 121 (30.4) | 107 (29.9) | **<0.001** |
| ARA, n (%) | 27 (2) | 374 (21.4) | 2 (0.8) | 6 (1.2) | 11 (3.3) | 8 (2.7) | 0.067 |
| ACA, n (%) | 508 (31.6) | 141 (8.1) | 64 (22.8) | 197 (32.5) | 126 (33.1) | 121 (35.7) | **0.004** |
| Therapies |  |  |  |  |  |  |  |
| Ongoing bDMARDs, n (%) | 94 (5.4) | 0 | 32 (10.3) | 38 (5.7) | 15 (3.7) | 10 (2.7) | **<0.001** |
| Ongoing csDMARDS, n (%) | 577 (33) | 0 | 108 (34.7) | 228 (34.4) | 137 (33.7) | 105 (28.6) | 0.234 |
| Ongoing Ccs, n (%) | 631 (36.1) | 0 | 109 (35) | 228 (34.4) | 167 (41) | 128 (34.9) | 0.138 |
| Ongoing ERA, n (%) | 399 (22.8) | 0 | 98 (31.5) | 167 (25.2) | 76 (18.7) | 58 (15.8) | **<0.001** |
| Ongoing PDE5, n (%) | 79 (4.5) | 0 | 16 (5.1) | 29 (4.4) | 20 (4.9) | 14 (3.8) | 0.837 |
| Ongoing Prost, n (%) | 995 (57) | 0 | 204 (65.6) | 406 (61.2) | 228 (56) | 158 (43.1) | **<0.001** |

Table 2 Supplementary: General characteristics according to age at NRP first sign/symptom

*NRP non-Raynaud’s phenomenon, RP Raynaud’s phenomenon, SD standard deviation, n numbers, y years, IQR Interquartile Range, BMI Body Mass index, CCI Charlson comorbidity index, , dcSSc diffuse cutaneous systemic sclerosis, , mRSS modified Rodnan skin score, ILD interstitial lung disease, PAH pulmonary arterial hypertension, EF Ejection Fraction, sPAP Systolic Pulmonary Artery Pressure, FVC Forced Vital Capacity, DLCO Diffusing Capacity of the Lung for Carbon Monoxide, ANA antinuclear antibodies, ATA anti-topisomerase antibodies, ARA Anti-RNA polymerase III antibodies, ACA anti-centromere antibodies, bDMARDs Biologic Disease-Modifying Antirheumatic, csDMARDS Conventional Synthetic Disease-Modifying Antirheumatic Drugs, Ccs Corticosteroids, ERA Endothelin Receptor Antagonists, PDE5 Phosphodiesterase type 5 inhibitors, Prost Prostanoids,*

|  |  | β | 95% CI  Lower Bound | 95% CI  Upper Bound | P-value |
| --- | --- | --- | --- | --- | --- |
| *DLCO* | RP first | 0.928 | -1,898 | 3.753 | 0.520 |
| *1095/1748(62.6%)* | age_RP | 0.039 | -0.146 | 0.224 | 0.678 |
| *patients included* | (Constant) | 88.419 | 82.559 | 94.278 | <0.001 |
| *sPAP* | RP first | -0.872 | -3.117 | 1.373 | 0.446 |
| *1218/1748(69.6%)* | age_RP | -0.149 | -0.297 | -0.001 | 0.049 |
| *patients included* | (Constant) | 5.550 | 1.224 | 9.876 | 0.012 |
| *FVC* | RP_first | 2.722 | -0.216 | 5.659 | 0.069 |
| 1148/1748(65.6%) | age_RP | 0.083 | -0.112 | 0.278 | 0.405 |
| *patients included* | (Constant) | 96.078 | 89.897 | 102.260 | <0.001 |
| *mRSS* | RP_first | -0.463 | -1.082 | 0.156 | 0.142 |
| 1572/1748(89.9%) | age_RP | 0.083 | -0.112 | 0.278 | 0.405 |
| *patients included* | (Constant) | 96.078 | 89.897 | 102.260 | <0.001 |

Table 3 Supplementary: Linear regression in a multivariate analysis. All factors are adjusted for covariates.

*RP Raynaud’s phenomenon, DLCO Diffusing Capacity of the Lung for Carbon Monoxide, sPAP Systolic Pulmonary Artery Pressure, TLC Total Lung Capacity, FVC Forced Vital Capacity,* *mRSS modified Rodnan skin score.*

|  | Item No | Recommendation | Page No |
| --- | --- | --- | --- |
| **Title and abstract** | 1 | (a)Indicate the study’s design with a commonly used term in the title or the abstract  YES The title specifies the design as a retrospective, multicenter cross-sectional study (a retrospective analysis from the Italian national multicenter SPRING (Systemic Sclerosis Progression InvestiGation) Registry of the Italian Society for Rheumatology) | 1 |
|  |  | (*b*) Provide in the abstract an informative and balanced summary of what was done and what was found  YES The abstract summarizes background, study design, patient population, methods, key findings, and conclusions. | 4-5 |
| Introduction | | | |
| Background/rationale | 2 | Explain the scientific background and rationale for the investigation being reported  YES Background on RP/NRP onset patterns in SSc and rationale for study. | 6 |
| Objectives | 3 | State specific objectives, including any prespecified hypotheses  YES. Objectives stated in final paragraph of Introduction (impact of RP/NRP onset timing and age on disease features/outcomes). | 6 |
| Methods | | | |
| Study design | 4 | Present key elements of study design early in the paper  YES.Retrospective, multicenter, registry-based, cross-sectional.Present key elements of study design early in the paper | 7 |
| Setting | 5 | Describe the setting, locations, and relevant dates, including periods of recruitment, exposure, follow-up, and data collection  YES. Patients were enrolled in the SPRING registry, a multicenter, national cohort study promoted by the Italian Society of Rheumatology (SIR) which involved 38 referral centers across Italy, with expertise in the diagnosis and management of SSc. | 7 |
| Participants | 6 | Give the eligibility criteria, and the sources and methods of selection of participants  YES. Inclusion/exclusion criteria detailed; | 7-8 |
| Variables | 7 | Clearly define all outcomes, exposures, predictors, potential confounders, and effect modifiers. Give diagnostic criteria, if applicable  YES RP/NRP onset definitions, clinical variables, autoantibodies, organ involvement. | 8-9 |
| Data sources/ measurement | 8* | For each variable of interest, give sources of data and details of methods of assessment (measurement). Describe comparability of assessment methods if there is more than one group  YES Data were extracted from the SPRING registry. Clinical and laboratory assessments followed standardized definitions (mRSS, DLCO, FVC, echocardiography, HRCT, serology). Same definitions applied across all onset groups, ensuring comparability. | 8-9 |
| Bias | 9 | Describe any efforts to address potential sources of bias  YES. Multivariable regression models adjusted for potential confounders. Selection bias and possible misclassification of onset timing are acknowledged in the Limitations section. | 10,25 |
| Study size | 10 | Explain how the study size was arrived at  YES. No a priori sample size calculation; all eligible patients in registry were included. | 10 |
| Quantitative variables | 11 | Explain how quantitative variables were handled in the analyses. If applicable, describe which groupings were chosen and why  YES. Age of onset grouped according to cohort distribution; continuous variables summarized as mean/SD or median/IQR. | 9-10 |
| Statistical methods | 12 | (a)Describe all statistical methods, including those used to control for confounding  YES. Multinomial regression, Cox regression, adjusted models. | 9-10 |
|  |  | (*b*) Describe any methods used to examine subgroups and interactions  YES. Subgroup analysis by onset pattern (RP, Simultaneous, NRP) | 8 |
|  |  | (*c*) Explain how missing data were addressed  YES. Missing data excluded from regression models; acknowledged in Limitations. | 10 |
|  |  | (*d*) If applicable, describe analytical methods taking account of sampling strategy  Not applicable (registry-based). | - |
|  |  | (*e*) Describe any sensitivity analyses  Not performed | - |
| Results | | | |
| Participants | 13* | (a)Report numbers of individuals at each stage of study—eg numbers potentially eligible, examined for eligibility, confirmed eligible, included in the study, completing follow-up, and analysed  YES Total n and excluded patients reported in Results | 10 |
|  |  | (b) Give reasons for non-participation at each stage  YES. Exclusion criteria described. | 8 |
|  |  | (c) Consider use of a flow diagram  Not included (narrative description and Table given) | 11-14 |
| Descriptive data | 14* | (a)Give characteristics of study participants (eg demographic, clinical, social) and information on exposures and potential confounders  YES.Provided in Table 1 | 11-13 |
|  |  | (b) Indicate number of participants with missing data for each variable of interest.  YES. Provided in Table 1 | 11-13 |
| Outcome data | 15* | Report numbers of outcome events or summary measures  YES. Survival and mortality events reported | 17-19 |
| Main results | 16 | 1. Give unadjusted estimates and, if applicable, confounder-adjusted estimates and their precision (eg, 95% confidence interval). Make clear which confounders were adjusted for and why they were included.   YES. Multivariable models reported with adjusted HR/OR and 95% CI. | 14-18 |
|  |  | 1. Report category boundaries when continuous variables were categorized   YES. Age of onset thresholds specified | 13-14 |
|  |  | 1. If relevant, consider translating estimates of relative risk into absolute risk for a meaningful time period   Not applicable | - |
| Other analyses | 17 | Report other analyses done—eg analyses of subgroups and interactions, and sensitivity analyses  YES. Multinomial regression was performed | 16-17 |
| Discussion | | | |
| Key results | 18 | Summarise key results with reference to study objectives.  YES. Key findings summarized in first paragraph of Discussion. | 19-20 |
| Limitations | 19 | Discuss limitations of the study, taking into account sources of potential bias or imprecision. Discuss both direction and magnitude of any potential bias  YES. Dedicated Limitations subsection covers design, misclassification, small NRP group, lack of power analysis, absence of longitudinal data. | 25 |
| Interpretation | 20 | Give a cautious overall interpretation of results considering objectives, limitations, multiplicity of analyses, results from similar studies, and other relevant evidence  YES. Interpretation integrates present data with Spanish, EUSTAR, and antisynthetase studies. | 20-22 |
| Generalisability | 21 | Discuss the generalisability (external validity) of the study results.  YES. Clinical and external validity are well discussed. | 23-24 |
| Other information | | | |
| Funding | 22 | Give the source of funding and the role of the funders for the present study and, if applicable, for the original study on which the present article is based  YES. Funding statement included in Funding section | 27 |

Table 4 Supplementary: STROBE Statement Checklist of items for cross-sectional studies
